# Chemistry of the Four Seasons—Spring, Summer, Autumn, and Winter: An Essay Principally Concerning Natural Phenomena, Admitting of Interpretation by Chemical Science, and Illustrating Passages of Scripture

**Published:** 1847-02

**Authors:** 


### ARTICLE XII.

*Chemistry of the Four Seasons—Spring, Summer, Autumn, and Winter: An Essay principally concerning Natural Phenomena, admitting of interpretation by Chemical Science, and illustrating passages of Scripture.* By THOMAS GRIFFITHS,

Professor of Chemistry in the Medical College of St. Bartholomew's Hospital, &c. &c. Philadelphia: Lea & Blanchard. 1846. pp. 451. 12mo. (From the Publishers. For sale by Brautigam & Keen, Chicago.)

This little work is founded upon Lectures, delivered by the author at scientific institutions in London and Liverpool, and is an exceedingly comprehensive epitome of chemical science, as connected with natural phenomena. The author has succeeded to a remarkable degree, in the difficult task of popularizing science, and whilst his production is rendered perfectly comprehensible to the uninitiated, for whom expressly it was written, it cannot be said to be superficial. We cannot but admire the aptness of the author's experimental illustrations, and their perfect simplicity; so simple and easy of performance, indeed, are they, that they may be repeated successfully by the merest tyro in chemical manipulations. The style of the work is unusually agreeable and attractive, and to the serious it will recommend itself by the beauty and frequency of its reference to the evidences of design in the phenomena of nature. The part of the work describing the properties and modes of examining soils chemically, the influence of light, heat, electricity, &c., upon vegetation, will render the work valuable and interesting to horticulturists and husbandmen. In a word, no one can read this admirable little book without both pleasure and profit. The mechanical execution of the American edition does credit even to its well known publishers. J. V. Z. B.

